# On the Clinical Manifestations of Rotheln, or German Measles

**Published:** 1885-11-20

**Authors:** W. A. Edwards

**Affiliations:** Assistant Demonstrator of Clinical Medicine, University of Pennsylvania


### ON THE CLINICAL MANIFESTATIONS OF ROTHELN, OR GERMAN MEASLES.

BY W. A EDWARDS, M. D.,

Assistant Demonstrator of Clinical Medicine, University of Pennsylvania.

The followiug statements are based upon the observation of about ten cases of Rotheln, of which some hundred odd cases were admitted to the Philadelphia Hospital in 1881-'82.

### STAGE OF INCUBATION.

This is, of course, the most difficult to decide positively, as symptoms were entirely absent during this time. As near as we could ascertain, the duration of this stage was about ten days; at all events, between ten and twelve. The shortest period recorded was six days, and longest twenty-one.

### STAGE OF INVASION—SYMPTOMS.

Chilliness, languor, faintness, headache more or less severe, pain in the back and limbs, coryza, red and watery eyes, *sore throat*, cough, and a hoarse, husky voice are the most frequent notes of this stage. The following additional symptoms were noted in 15 per cent. of the cases: nausea and vomiting, delirium and convulsions, and epistaxis in three cases. Two cases of hemorrhage from the eyes and ears are recorded by J. Ford Prioleau. The average duration of this stage, in our cases, was three days, when the eruption appeared, the general symptoms in the meanwhile incresing in severity.

### ERUPTIVE STAGE—SYMPTOMS.

After the above-noted symptoms had lasted about three days, an eruption appeared, generally first in the face, and rapidly extending over the whole body; the existing symptoms were greatly aggravated, in fact beyond what the temperature and pulse would seem to indicate. Occasionally a prodromal rash was noted to precede the specific eruption, more marked in the buccal cavity. C. J. Collingworth has four times seen a severe attack of urticaria precede, for a few hours, the development of the characteristic rash of Rotheln. Erythema preceded the exanthem in four of our cases. Rapid extension of the eruption progressively downward, about in the following order, was most frequently noted: face, neck, chest, arms, back, groin, and lower extremities. The rash was multiform in character, more or less confluent, occasionally ill-defined; in color, rosy or pale red. A few cases of the brightest scarlet and purplish tints were observed. Patterson, of Leith, compares the appearance of the patch in color to that "produced by a writing-quill dipped in red ink, and having its point placed on moist white paper." The rash was punctuated, small macules were noted; over the non-vascular parts the rash was elevated, producing a rough skin easily detected by the touch. The patches were very irregular in outline, shape, and size, the factor being the most irregular. The centre of each patch was much higher in color than any other part. Much hyperæmia of the intervening skin was present in many cases ; itching was then a more marked symptom.

Dunlop says that he has seen petechiæ, as has also J. L. Erskine, in the uvula and soft palate. In rare instances the eruption goes on to the formation, upon hyperæmic spots, of a varying number of vesicles resembling miliaria. Reed records a case in which the eruption alone appeared on the tonsils and velum palati, but no rash whatever on any external part of the body. According to Thomas, the eruption is due to a capillary hyperæmia of the papillary body of the uppermost layers of the corium. The eruption was generally discrete ; had little tendency to become confluent ; when it did occur, it was most marked on the face or extremities.

Superadded to the previously existing symptoms, the eruptive stage presented rise in temperature of from 1 to 3 degrees ; 103°, 104 are recorded in the notes, the temperature being in proportion to the extent and severity of the eruption of the chest. Sore throat was *always* present, with enlargement of the tonsils, in some cases to a great extent. Enlargement and induration of the cervical, post cervical, and post auricular glands were now present. Occasonally only one or two glands would be affected ; in other cases the entire chin. The cough was generally increased in severity and frequency, and became more laryngeal. In quite a fair proportion of cases vomiting occurred as the eruption was approaching its maximum ; in five cases it was almost uncontrollable.

PULSE.

The pulse-respiration ratio was in all the cases maintained, it falling with the temperature, and that with the disappearance of the rash. Pulses of 120, 130, 140, 150 were recorded. Several cases presented well-marked symptoms of heart failure, but were successfully treated by general and cardiac tonics.

TONGUE.

Coated as the scarlatinous tongue, but never exfoliated as the tongue does in that disease. The "strawberry" tongue was never met with ; dry brown tongue appears in the notes of the more severe cases. Cleaning in patches was the most usual method of return to the normal appearance.

URINE.

This secretion was such as is found in all similar states, "febrile urine." Slight albuminuria was present in about 50 per cent. of the cases. Nine cases presented well marked albuminous urine (one-fifteenth bulk) with dropsy. No tube casts, however, could be detected.

DURATION AND TERMINATION OF THE ERUPTION.

The average duration was five days ; the shortest was scarcely two days, and the longest of all the cases was fifteen. The eruption in all the cases was followed by desquamation of furfuraceous scales. In quite a number of the cases the desquamation was well marked, in others only on particular parts of the body ; in these instances especially about the nose. A delicate brownish-yellow pigmentation was not infrequently observed after the eruption had subsided ; this coloring did not appear to bear any relation to the color of the eruption or its severity. The buccal cavity also partook of the general desquamation ; it was here best marked in the throat proper.—*American Journal of Medical Sciences.*

